# Assessing the spatiotemporal dynamics and driving factors of human brucellosis in Northern Xinjiang, China (2015–2023)

**DOI:** 10.1186/s41182-026-00899-6

**Published:** 2026-01-17

**Authors:** Peiyao Zhou, Liping Zhang, Feifei Li, Xiaodong Wang, Jiangshan Zhao

**Affiliations:** 1 Institute of Medical Engineering Interdisciplinary Research, College of Medical Engineering and Technology, Xinjiang Medical University, Urumqi, 830017 China; 2 Xinjiang Uygur Autonomous Region Center for Disease Control and Prevention, Urumqi, 830017 Xinjiang China

**Keywords:** Brucellosis, MGWR, JPR, Spatial epidemiology

## Abstract

**Objectives:**

Brucellosis has long been a major public health concern in western pastoral areas. This study aimed to explore the spatiotemporal dynamic evolution of human brucellosis in northern Xinjiang and analyze the spatial heterogeneity of factors influencing its incidence in different counties and districts.

**Methods:**

The JPR (Joinpoint regression model) and spatial autocorrelation analysis were employed to capture the potential spatiotemporal distribution changes of human brucellosis in northern Xinjiang from 2015 to 2023. Multiscale Geographically Weighted Regression (MGWR) was established to analyze the spatiotemporal correlation between the incidence of human brucellosis and relevant influencing factors.

**Results:**

The incidence of brucellosis in northern Xinjiang from 2015 to 2023 showed a trend of first decreasing and then increasing, with a turning point in 2020. The incidence decreased from 47.92 per 100,000 in 2015 to 14.20 per 100,000 in 2020, with an average annual decrease of 21.68%. Subsequently, it increased to 38.70 per 100,000 in 2023, with an average monthly increase of 39.97%. The results of the MGWR model indicated that a higher regional gross output value of animal husbandry was associated with a higher incidence of human brucellosis, which was more prominent in Hami City and Turpan City. Regions with longer sunshine hours had a higher incidence of brucellosis, especially in the Ili Kazakh Autonomous Prefecture and Bortala Mongolian Autonomous Prefecture. A higher beef production was related to a higher incidence of human brucellosis, which was more evident in Turpan City, Hami City, and the Bayingolin Mongolian Autonomous Prefecture. A higher average regional temperature was associated with a higher incidence of human brucellosis. An increase in GDP had a significant protective effect on the incidence of human brucellosis.

**Conclusions:**

Human brucellosis remains a serious problem in northern Xinjiang, and its incidence has been on the rise in recent years. Meteorological and social factors influence the incidence of brucellosis spatially heterogeneously, with the direction and magnitude of their effects varying significantly across specific geographic locations in Northern Xinjiang. Consequently, we recommend a shift from uniform policies to zonal management, prioritizing interventions based on local dominant risk factors (e.g., sheep population control in Bayingolin, occupational protection in high-livestock-production areas) to optimize resource allocation and control effectiveness.

**Supplementary Information:**

The online version contains supplementary material available at 10.1186/s41182-026-00899-6.

## Introduction

Brucellosis is a zoonotic disease caused by gram-negative bacteria of the genus Brucella. It is globally distributed, with significant endemicity in regions characterized by intensive livestock production [1]. Epidemiological estimates indicate around 500,000 new human cases annually worldwide. High-prevalence areas include the Mediterranean Basin, the Middle East, Central Asia, Latin America, and sub-Saharan Africa [2]. The spatial distribution of brucellosis is strongly linked to regions, where agricultural practices and consumption of unpasteurized dairy products elevate the risk of animal-to-human transmission [3]. In China, brucellosis remains a major public health challenge, particularly in northern pastoral and semi-agricultural regions, such as Inner Mongolia, Xinjiang, and Qinghai, where reported incidence is substantially higher than in other parts of the country [4, 5]. National surveillance data consistently rank China among the countries with the highest human brucellosis caseloads globally. A notable resurgence over the past two decades underscores its status as a re-emerging endemic disease [6, 7]. Northern Xinjiang, in particular, is recognized as a hyperendemic zone. The region’s arid climate, marked by extreme diurnal temperature variation, may facilitate environmental persistence and transmission of Brucella. Moreover, the local economy relies heavily on livestock farming, and frequent close contact between humans and animals—especially sheep, cattle, and goats—further heightens the risk of zoonotic spillover [8, 9].

Accumulating evidence highlights the influence of climatic factors on brucellosis epidemiology [10]. Factors, such as temperature, humidity, and precipitation, not only affect the survival and dissemination of the pathogen in the environment but may also indirectly influence transmission dynamics through changes in host behavior, livestock management practices, and human–animal interactions [11]. Studies suggest that warm and humid microclimates can prolong the extracellular viability of Brucella in fomites, while climate extremes such as droughts or floods may disrupt the wildlife–livestock–human interface and modify anthropogenic activities, thereby altering transmission cycles [12, 13]. Brucellosis exhibits considerable spatiotemporal heterogeneity, a key epidemiological feature of the disease [14–16]. Temporally, incidence follows distinct seasonal patterns, with peaks typically occurring in spring and summer, coinciding with livestock reproductive cycles, hormonal changes, and intensified human–livestock contact during farming activities [17]. Geographically, the disease displays marked disparities, with hyperendemic foci concentrated in areas of intensive animal husbandry, favorable climatic conditions, and specific topographic profiles [18].

This observed variation arises from complex interactions between environmental determinants—including temperature, solar radiation, and rainfall—and socioeconomic factors, such as livestock density, farming practices, public health infrastructure, and access to veterinary services [19]. Understanding these spatiotemporal patterns and their drivers is essential for formulating evidence-based, adaptive control strategies that address both ecological and anthropogenic risks.

To systematically characterize epidemiological trends of brucellosis in northern Xinjiang and their underlying determinants, this study employed an integrated analytical framework combining Joinpoint Regression Analysis and Multiscale Geographically Weighted Regression. The Joinpoint model was used to identify significant inflection points in temporal incidence trends, quantify long-term changes, and pinpoint critical historical transitions in disease dynamics [20]. This approach provides a scientific basis for designing time-sensitive interventions aligned with observed trend shifts. Complementing the temporal analysis, the MGWR model accounts for spatial non-stationarity, allowing detection of geographically varying relationships between climatic variables and brucellosis incidence [21, 22]. By incorporating scale-dependent parameter estimation, this technique reveals fine-grained spatial heterogeneity in risk-factor associations, distinguishing between broad regional climatic influences and localized anthropogenic effects. Through the synergistic application of these two frameworks, this study aims to delineate the spatiotemporal dynamics of brucellosis in northern Xinjiang, quantify bidirectional interactions between climatic factors and disease transmission, and identify priority intervention zones based on geographically differentiated risk profiles. The findings are intended to inform evidence-based policy, supporting targeted and sustainable interventions to reduce incidence and transmission risks at both regional and local scales [23]. By providing actionable insights into spatiotemporal patterns and their drivers, this study may aid in developing precision public health measures to mitigate transmission and lower disease burden in this vulnerable region.

## Materials and methods

### Study area

The study area encompasses the northern region of the Xinjiang Uygur Autonomous Region in China, including the following major areas: Urumqi City, Turpan City, Hami City, Karamay City, Altay Prefecture, Tacheng Prefecture, Changji Hui Autonomous Prefecture, Ili Kazakh Autonomous Prefecture, Bortala Mongol Autonomous Prefecture and Bayingolin Mongol Autonomous Prefecture. This region is located in the northwestern part of China, with approximate latitude and longitude ranges of 42°N–49°N and 80°E–95°E. It borders Kazakhstan, Russia, and Mongolia, making it a geographically significant area.

Northern Xinjiang features diverse topography: the eastern part consists of vast Gobi deserts, while the western part is home to the fertile Ili River Valley. The Tianshan Mountains run east–west, dividing Xinjiang into northern and southern parts: the Junggar Basin in the Northern and the Tarim Basin in the South. The Altai Mountains in the Northern form a natural boundary with Mongolia and Russia. The region's complex terrain includes high mountains, deep valleys, expansive grasslands, and arid deserts, contributing to its remarkable ecological diversity. The climate is characterized by warm to hot summers, with temperatures typically ranging from 20 to 35 °C, and cold winters, especially in high-altitude areas, where temperatures can drop below −20 °C. Precipitation is scarce, with most areas receiving less than 200 mm annually, primarily as snowfall in winter. The region experiences strong winds, particularly in desert areas, leading to frequent sandstorms.

### Data sources

The incidence data of human brucellosis in northern Xinjiang from 2015 to 2023 were obtained from the Xinjiang Uygur Autonomous Region Centers for Disease Control and Prevention. This study determined suspected and confirmed cases based on the "Diagnostic Criteria for Brucellosis (WS 269-2019)" of China. Meteorological factors (such as annual average temperature, humidity, and precipitation) and socio-economic factors (including year-end cattle and sheep populations, beef and mutton production, total livestock output value, and regional GDP) were sourced from the Xinjiang Statistical Bureau. Geographic information was obtained from the National Geomatics Center of China (http://www.ngcc.cn/). The map of China used in this study was based on a standard map (approval number: GS (2024)0650) downloaded from the Standard Map Service website of the Ministry of Natural Resources (http://www.bzdt.ch.mnr.gov.cn/).

In this study, collinearity diagnostics were performed on 12 key variables: Gross Regional Product (GRP), Gross Primary Product of animal husbandry (GPP), Annual Sunshine Hours (ASH), Annual Average Temperature (AAT), Beef Production (BP), Mutton Production (MP), Minimum Temperature (TMIN), Annual Precipitation (AP), Maximum Temperature (TMAX), Total Livestock Quantity (TN), Number of Cattle Raised (NOC), and Number of Sheep Raised (NOS). Variables exhibiting a variance inflation factor exceeding 10 were sequentially eliminated from the analysis. Following this screening process, 8 variables (GRP, GPP, ASH, BP, MP, TMIN, AP, and NOC) were retained for inclusion in the final predictive model. TMAX and NOS raised were retained in the model despite elevated VIF, as they are recognized critical risk factors for brucellosis, and their inclusion was deemed essential for the model's contextual validity.

### Model introduction

#### Model selection

The traditional generalized linear model assumes spatial homogeneity and is unable to capture the relationships of driving factors that vary with geographical locations. Although machine learning (ML) and deep learning (DL) models may have advantages in prediction accuracy, their "black box" nature makes the interpretability of results poor. The main goal of this study is to explain the spatiotemporal heterogeneity of driving factors rather than merely making predictions. The core advantage of the MGWR model lies in its ability to reveal the regression coefficients of each independent variable in different geographical units, thereby intuitively demonstrating the variation of their spatial effects, whereas the JPR model is adept at handling the spatio-temporal dependencies in panel data. Therefore, these two models are more targeted and interpretable in achieving the core objective of this study than GLM, ML, or DL.

#### Joinpoint model

The joinpoint regression model was constructed to analyze the temporal characteristics of human brucellosis prevalence across different regions. This model allows for the identification of inflection points (joinpoints), which determine the locations and directions of change in the incidence trend. The Grid Search Method was employed to select the optimal joinpoints. Specifically, the parameter space was divided into a grid, and each intersection point represented a potential solution [24]. Within a defined interval, the performance metrics of each solution were calculated step-by-step at fixed intervals to determine the optimal parameters. For the joinpoint model analysis, the GSM generated a grid covering all possible joinpoint locations, computed the sum of squared errors (SSE) and mean squared errors (MSE) for each scenario, and selected the grid with the smallest MSE as the optimal joinpoint [25]. The annual percentage change (APC) and average annual percentage change (AAPC) were then calculated to clarify the direction and rate of change in both the overall and local regions. When the APC is greater than 0, the incidence rate shows an increasing trend year by year; conversely, when the APC is less than 0, the incidence rate shows a decreasing trend [26]. The formulas for APC and AAPC are as follows:

1 $$ {\mathrm{APC}}\,{ = }\,\left\{ {\exp \,\left( {\upbeta } \right)\, - \,1} \right\}\, \times \,100 $$

2 $$ {\mathrm{AAPC}}\,{ = }\,\left\{ {\exp \,\left( {\frac{{\sum {{\mathrm{w}}_{{\mathrm{i}}} \beta_{{\mathrm{i}}} } }}{{\sum {{\mathrm{w}}_{{\mathrm{i}}} } }}} \right)\, - \,1} \right\}\, \times \,100, $$

where *β* is the regression coefficient,${w}_{i}$ is the number of years included in each paragraph, and $\beta_{i}$ is the regression coefficient of each paragraph.

#### Multi-scale geographical weighted regression model

The application of spatial econometric methods assumes spatial heterogeneity in sample data [27]. Therefore, before constructing the MGWR model, spatial autocorrelation analysis was performed on the independent variables [28]. Typically, Moran's I was used for global spatial autocorrelation analysis to determine whether the attribute values of the studied sample points exhibited spatial correlation with those of other sample points in the domain [29]. A negative Moran's I index indicates that the attribute values between samples are discrete, whereas an index of 0 implies that the attribute values are randomly distributed without significant spatial characteristics. The absolute value of Moran's I represents the strength of the correlation; the larger the absolute value, the stronger the correlation. Mathematically, it is expressed as

3 $$ I\, = \,\frac{{\sum\nolimits_{i\, = \,1}^{n} {\sum\nolimits_{j\, = \,1}^{n} {\left( {x_{i} \, - \,\overline{x}} \right)\left( {x_{j} \, - \,\overline{x}} \right)} } }}{{S^{2} \,\sum\nolimits_{i\, = \,1}^{n} {\sum\nolimits_{i\, = \,1}^{n} {w_{ij} } } }} $$

where *I* is Moran's I value, *n* is the number of spatial units, ${x}_{i}$ and ${x}_{j}$ are the attribute values of spatial units *I* and *j*, $\overline{x }$ is the average attribute value of all study regions, and ${w}_{ij}$ is the spatial weight matrix between spatial units *I* and *j*.

The MGWR model is used to describe the differential scale effects of different independent variables, fully utilizing the spatial heterogeneity of sample data to improve the accuracy of parameter estimation [30]. The model is as follows:

4 $$ Y_{{\mathrm{i}}} \, = \,\beta_{0} \,\left( {u_{i} ,\,v_{i} } \right)\, + \,\sum\nolimits_{i\, = \,1}^{m} {\beta_{{{\mathrm{bw}}\, \cdot \,{\mathrm{k}}}} \,\left( {u_{i} ,\,v_{i} } \right)X_{ik} \, + \,\varepsilon_{i} ,} $$

where ${X}_{ik}$ i_s_ the dependent variable, $\left({u}_{i},\left.{v}_{i}\right)\right.$ is the independent variable at the sample point, ${\beta }_{k}\left({u}_{i},{v}_{i}\right)$ is the regression coefficient of the independent variable at location. ε is the random error. The differential bandwidth of the independent variables is represented by ${\beta }_{bw\cdot k}$. These parameters are estimated using weighted least squares. The MGWR model initially set the parameters as those of the GWR model and then corrected the model parameters using the back-fitting algorithm [31].

Based on the fitting coefficients of the MGWR model, the spatiotemporal distribution of each variable was plotted. The natural breaks method was used to group data with high similarity [32]. A positive fitting coefficient indicates that the independent variable has a promoting effect on the dependent variable, whereas a negative coefficient indicates an inhibitory effect. The larger the absolute value of the coefficient, the greater the degree of influence.

### Statistical analysis

The Joinpoint Regression Software (joinpoint Regression Program version 4.9.1.0) was used for time-trend analysis. ArcGIS (ArcGIs Desktop, version 10.8.1) and Multiscale Geographically Weighted Regression (MGWR, version 2.2) were employed for mapping, model construction, and parameter estimation. These software tools, developed by professional teams, ensure reliability and wide acceptance. Results were considered statistically significant at *p* < 0.05.

## Results

### Basic information

**Fig. 1:**
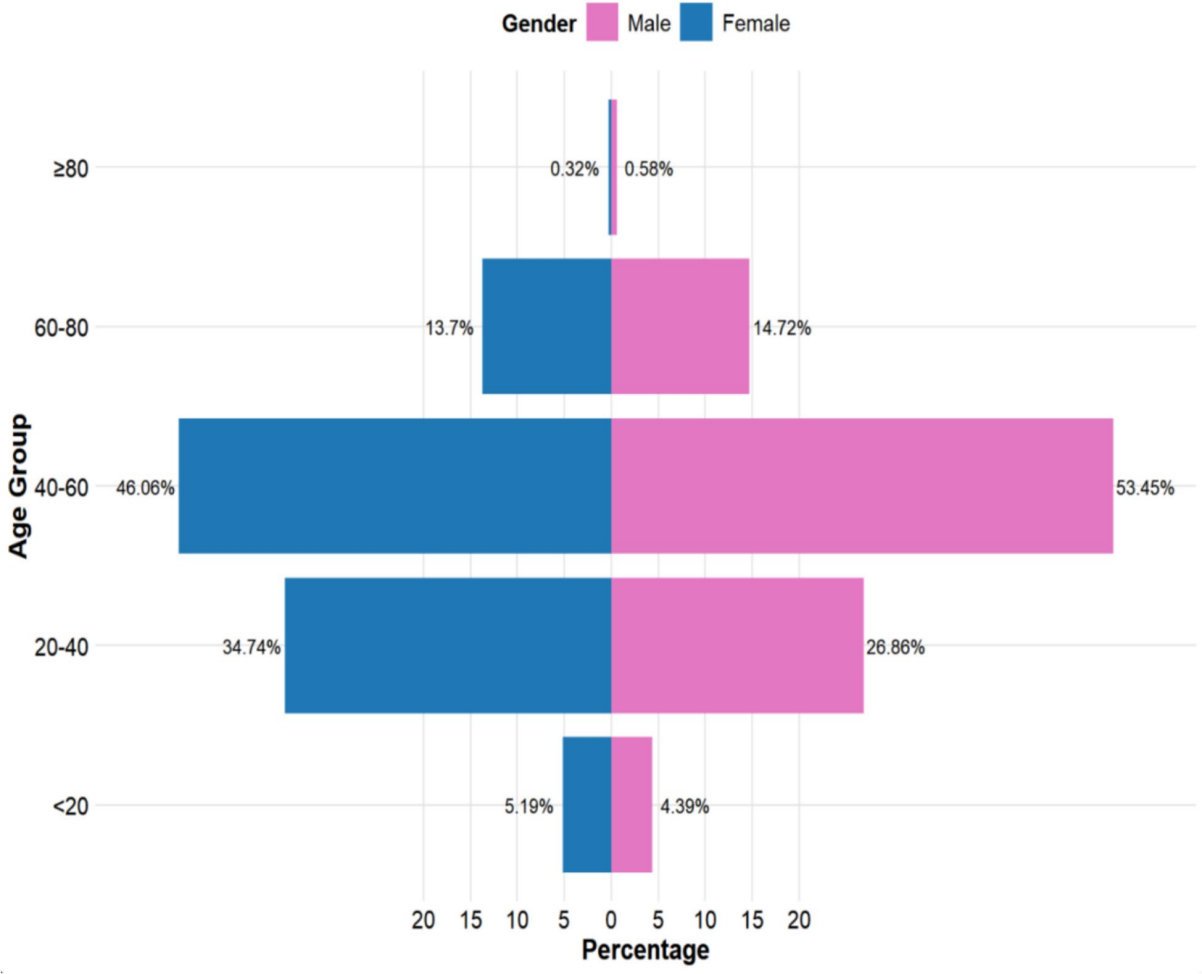
Analysis of the percentage distribution of brucellosis by age group and sex, 2015–2013

From 2015 to 2023, a total of 38,217 human brucellosis cases were reported in northern Xinjiang. Among them, 27,330 were male and 10,887 were female, accounting for 71% and 28% of the total, respectively. Among the 66 counties and districts, Qapqal Xibe Autonomous County, Huocheng County, and Qitai County had the highest number of cases, with 3,211, 2,704, and 1,998 cases, respectively, accounting for 8.4%, 7.1%, and 5.2% of the total. In terms of population classification, farmers, herders, and students had the most cases, with 26,442, 5,124, and 1,636 cases, respectively, accounting for 69%, 13%, and 4.3% of the total. The age distribution analysis shows that human brucellosis cases are mainly concentrated in the working-age population aged 40–59, and the number of cases in this age group accounts for 49.75% of the total reported cases. In the 40–60 age group, females exhibit a higher incidence rate than males, whereas in the 20–40 age group, males show a higher incidence rate than females. Further details are illustrated in Fig. 1.

### Trend analysis based on the Joinpoint model

#### Overall trend of brucellosis incidence in Northern Xinjiang

**Fig. 2:**
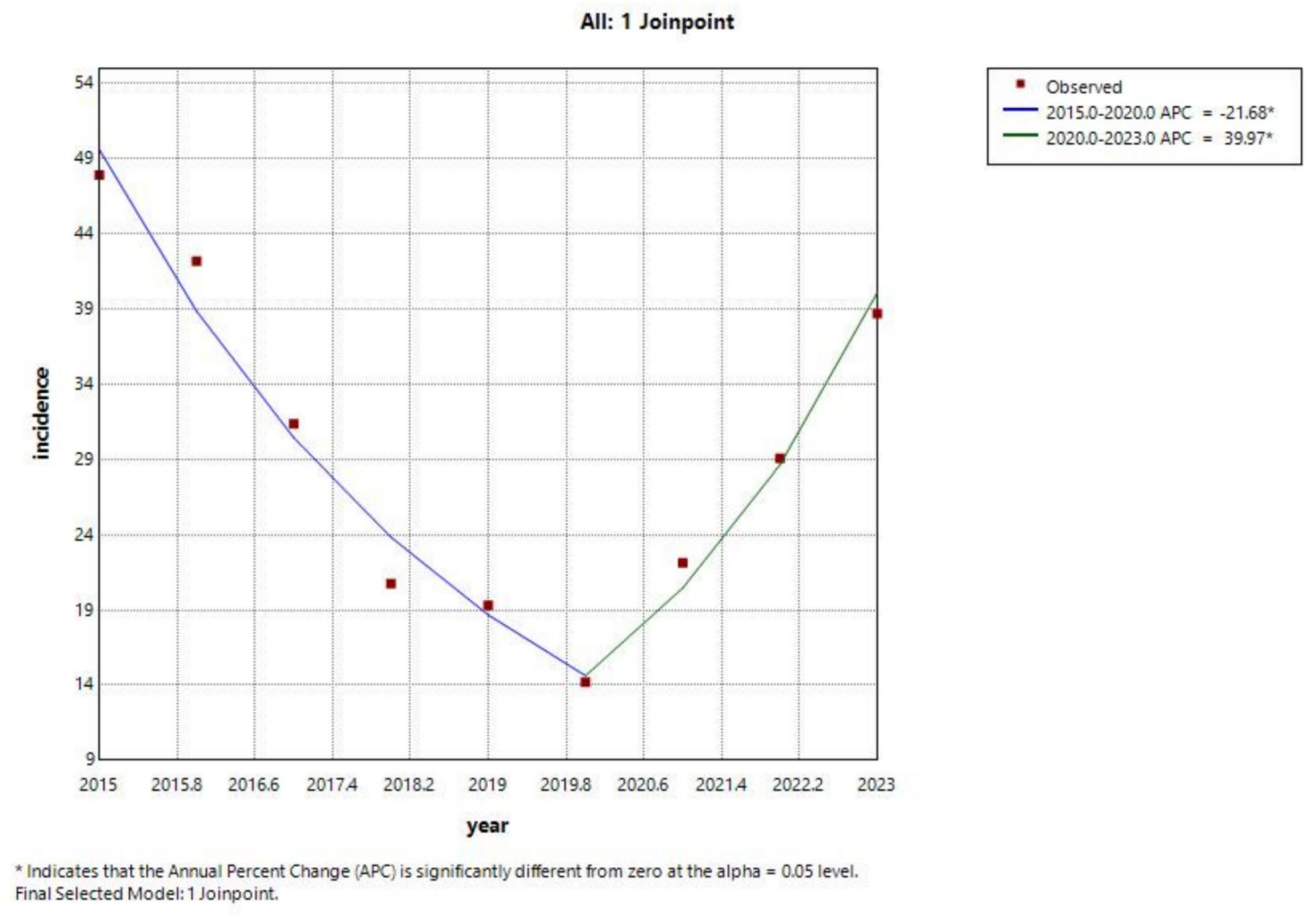
Overall trend of brucellosis incidence in northern Xinjiang from 2015 to 2023

The results of the Joinpoint regression model indicated that the incidence of brucellosis in northern Xinjiang from 2015 to 2023 showed a trend of first decreasing and then increasing, with a turning point in 2020. The incidence decreased from 47.92 per 100,000 in 2015 to 14.20 per 100,000 in 2020, with an average annual decrease of 21.68%. Subsequently, it increased to 38.70 per 100,000 in 2023, with an average monthly increase of 39.97%. However, the overall trend was not statistically significant (*p* > 0.05), see Table 1 and Fig. 2.

**Table 1:** Joinpoint regression model of the overall trend of brucellosis incidence in Northern Xinjiang from 2015 to 2023

Years	APC (95% CI%)	*p*	AAPC (95% CI%)	*p*
2015–2020	−21.6 (−26.6,−17.25)	< 0.05	−2.63 (−5.70,0.19)	0.15
2020–2023	39.97 (26.14,−65.41)	< 0.05		

APC represents annual percentage change; AAPC represents the average annual percentage change

#### Gender differences in brucellosis incidence trends

**Fig. 3:**
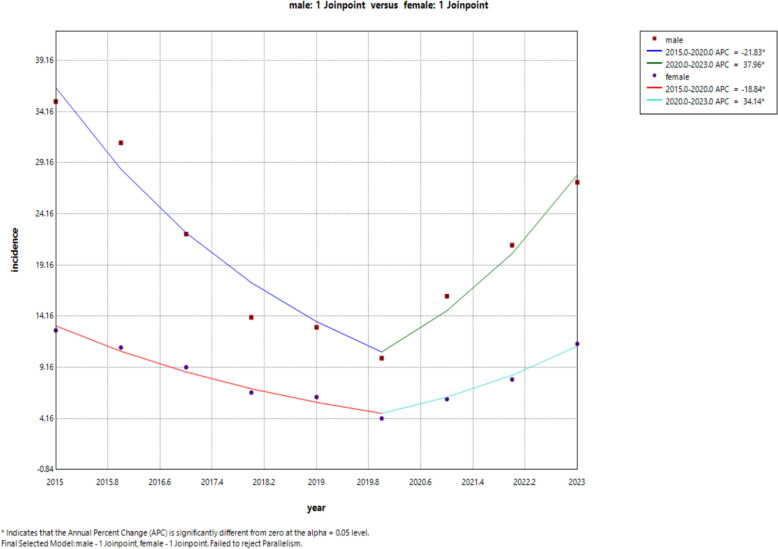
Gender trend of brucellosis incidence in northern Xinjiang from 2015 to 2023

The analysis of the Joinpoint regression model revealed that the incidence trends of brucellosis in both males and females demonstrated a pattern of initial decline followed by an increase, with a turning point in 2020, which was consistent with the overall trend. The female incidence rate decreased from 12.77 per 100,000 in 2015 to 4.16 per 100,000 in 2020, with an average annual decline of 21.83%. Subsequently, it increased to 11.45 per 100,000 in 2023, showing an average monthly increase of 37.96%. For males, the incidence decreased from 35.25 per 100,000 in 2015 to 10.04 per 100,000 in 2020, with an average annual decline of 18.84%, and then increased to 27.25 per 100,000 in 2023, with an average monthly increase of 34.14%. There was no significant difference in the overall trend between males and females (*p* > 0.05). Detailed information Detailed information can be found in Table 2 and Fig. 3.

**Table 2:** Joinpoint regression model of the gender trend of brucellosis incidence in northern Xinjiang from 2015 to 2023

Sex	Time (year)	APC (95% CI%)	*P*	AAPC (95% CI%)	*P*
Female	2015–2020	−21.83 (−28.64,−14.36)	< 0.05	−3.27 (−9.49,3.38)	0.32
	2020–2023	37.95 (12.93,68.52)	< 0.05		
Male	2015–2020	−18.83 (−22.34,−15.17)	< 0.05	−2.00 (−5.29,1.39)	0.24
	2020–2023	34.13 (20.66,49.11)	< 0.05		

APC represents annual percentage change; AAPC represents the average annual percentage change

#### Age differences in brucellosis incidence trends

The analysis of the Joinpoint regression model indicated that the incidence of brucellosis in the 0–79 age group exhibited a trend of initial decline followed by an increase, with a turning point in 2020, which was consistent with the overall trend. For the 80 + age group, the turning point occurred in 2021, and the incidence continued to rise from 2015 to 2023, showing a significant increase after 2021. The specific data are presented in Table 3.

**Table 3:** Joinpoint regression model of age trend of brucellosis incidence in Northern Xinjiang from 2015 to 2023

Cohort	Segment	Lower endpoint	Upper endpoint	APC (95% CI%)	*t*	*p*
< 19	1	2015	2020	−26.70 (−34.10,−18.46)	−8.10	< 0.05
< 19	2	2020	2023	51.69 (20.50,90.10)	5.03	< 0.05
20–39	1	2015	2020	−26.02 (−32.12,−19.39)	−9.73	< 0.05
20–39	2	2020	2023	30.57 (1.14,68.58)	2.89	< 0.05
40–59	1	2015	2020	−18.19 (−24.99,−10.78)	−6.42	< 0.05
40–59	2	2020	2023	37.82 (16.33,63.28)	5.25	< 0.05
60–79	1	2015	2020	−14.75 (−19.93,−9.24)	−7.07	< 0.05
60–79	2	2020	2023	36.71 (20.01,55.75)	6.66	< 0.05
≥ 80	1	2015	2021	0.64 (−12.13,15.27)	0.13	0.90
≥ 80	2	2021	2023	87.55 (27.88,175.08)	4.55	< 0.05

### Spatial autocorrelation analysis and MGWR model construction

#### Spatial autocorrelation analysis results

**Fig. 4:**
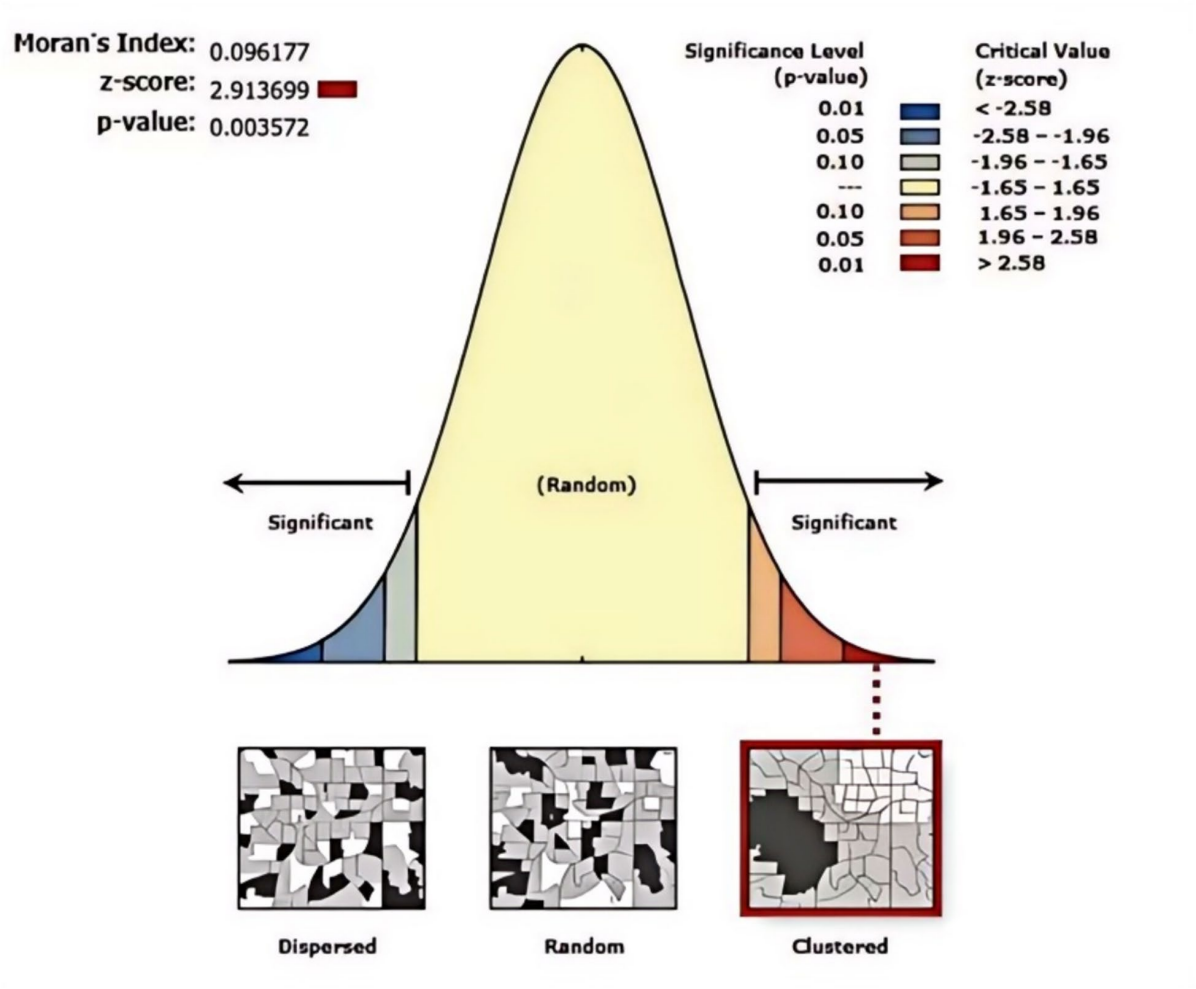
Spatial autocorrelation analysis of human brucellosis in northern Xinjiang

Spatial autocorrelation analysis showed that the Moran's I for human brucellosis incidence in northern Xinjiang from 2015 to 2023 was 0.096, indicating a positive spatial correlation. The Z-score was 2.913699 (*p* < 0.05), suggesting significant clustering (Fig. 4).

#### Spatiotemporal distribution of the MGWR regression coefficients

**Fig. 5:**
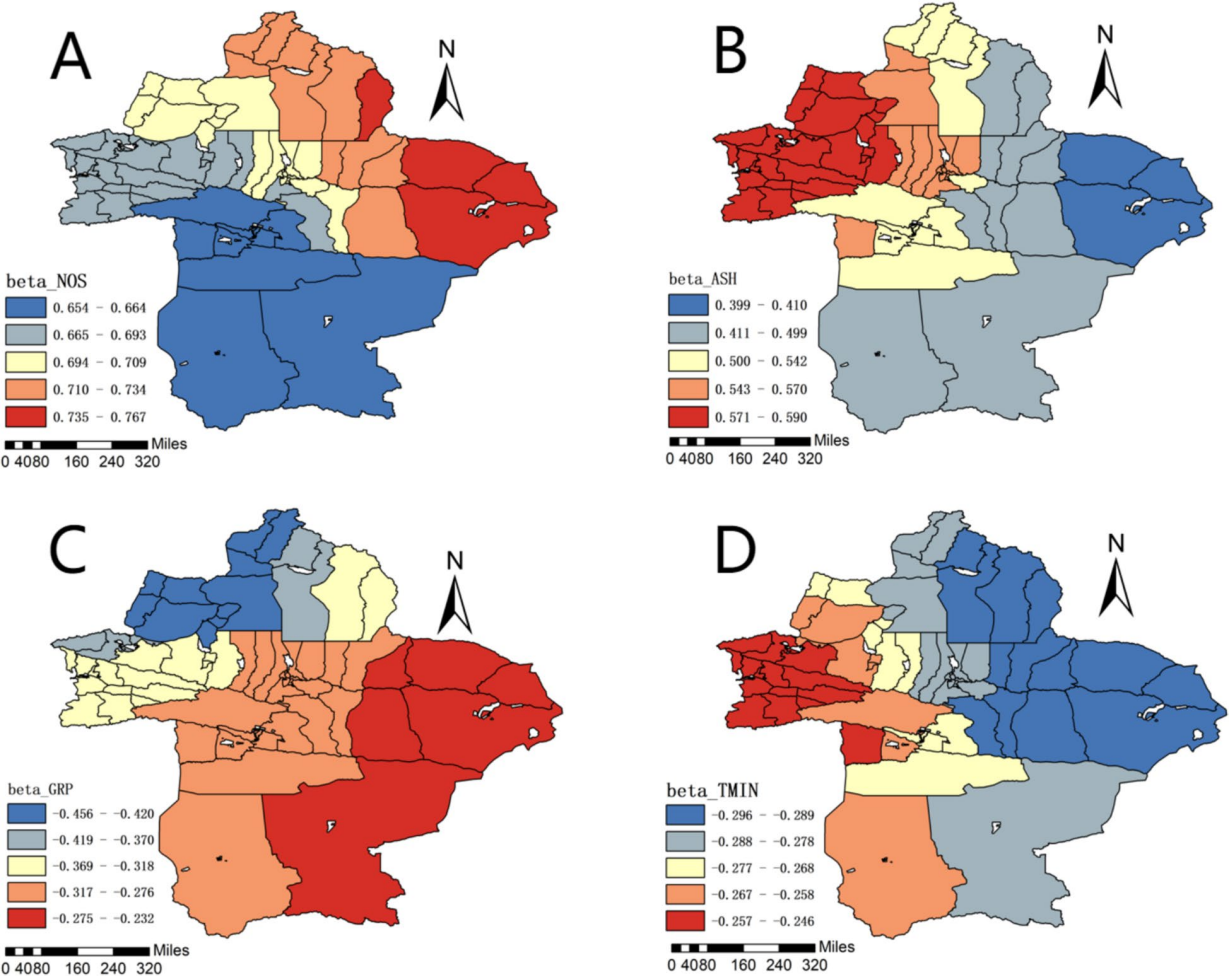
Spatial and temporal distribution of NOS, ASH, GRP, and TMIN regression coefficients

The parameter estimates of the MGWR model reflect the degree and direction of the influence of each factor on the incidence of brucellosis in different regions. The spatial distribution of the constant term represented the spatial variation in disease incidence under the "baseline level" of influencing variables, that is, factors not considered in this study. The coefficient estimates for GRP (regional GDP) were negative, indicating that a higher GDP was associated with a lower incidence of brucellosis, especially in Tacheng Prefecture and Altay Prefecture. The coefficient estimates for GPP (gross output value of animal husbandry) were positive, suggesting that a higher animal husbandry output was related to a higher incidence of brucellosis, particularly in Hami City and Turpan City. The coefficient estimates for ASH (annual sunshine hours) were positive, meaning that regions with longer sunshine hours had a higher incidence of brucellosis, especially in the Ili Kazakh Autonomous Prefecture and Bortala Mongolian Autonomous Prefecture. The coefficient estimates for BP (beef production) were positive, indicating that a higher beef production was associated with a higher incidence of brucellosis, which was more evident in Turpan City, Hami City, and the Bayingolin Mongolian Autonomous Prefecture. The coefficient estimates for TMIN (annual minimum temperature) were negative, showing that lower temperatures were related to a higher incidence of brucellosis, particularly in Altay Prefecture, Changji Hui Autonomous Prefecture, Turpan City, and Hami City. The coefficient estimates for AP (annual precipitation) were positive, suggesting that regions with more precipitation had a higher incidence of brucellosis. The coefficient estimates for TMAX (annual maximum temperature) were positive, indicating that a higher average temperature was associated with a higher incidence of brucellosis. The coefficient estimates for NOS (year-end sheep population) were positive, meaning that a higher sheep population was related to an increased incidence of brucellosis, especially in the Bayingolin Mongolian Autonomous Prefecture. The results are illustrated in Fig. 5, Table 4, and Supplementary Materials.

**Table 4:** Descriptive statistics of standardized regression coefficients for explanatory variables in the MGWR model

Variable	Mean	STD	Min	Median	Max	*P*
GRP (10,000 yuan)	−0.335	0.023	−0.456	−0.321	−0.232	< 0.05
GPP (10,000 yuan)	0.335	0.059	0.293	0.330	0.396	0.121
ASH (hours)	0.542	0.025	0.399	0.562	0.590	< 0.05
BP (tons)	0.180	0.050	0.147	0.171	0.238	0.860
MP (tons)	−0.284	0.024	−0.321	−0.289	−0.250	0.268
TMIN (℃)	−0.273	−0.013	−0.289	−0.274	−0.246	< 0.05
AP (mm)	0.440	0.077	0.345	0.421	0.593	0.093
TMAX (℃)	0.028	0.043	−0.032	0.035	0.091	0.743
NOC (10,000 heads)	−0.496	0.021	−0.516	−0.503	−0.433	0.059
NOS (10,000 heads)	0.696	0.024	0.654	0.695	0.767	< 0.05

*GRP* Gross regional product, *GPP* Gross output value of animal husbandry, *ASH* Annual sunshine hours, *BP* Beef production, *MP* Mutton production, *TMIN* Annual minimum temperature, *AP* Annual precipitation, *TMAX* Annual maximum temperature, *NOC* Year-end cattle population, *NOS* Year-end sheep population

## Discussion

Current research on brucellosis remains largely focused on descriptive epidemiology, encompassing areas, such as diagnosis, treatment, and immunization. However, brucellosis is a zoonotic disease influenced by a multitude of factors, including geographical and natural environments, local production and lifestyle practices, and the population size, distribution, and movement of infected livestock [33–35]. Therefore, it is of considerable importance to develop context-specific, precision prevention and control strategies tailored to local conditions.

Based on human brucellosis surveillance data from 2015 to 2023, Joinpoint regression analysis revealed that the incidence of brucellosis in Northern Xinjiang initially declined before rising again, with a turning point observed in 2020. As one of the China’s endemic regions, Xinjiang has implemented large-scale livestock vaccination programs against brucellosis since 2016, which contributed to a marked decrease in human incidence. Moreover, the stringent COVID-19 containment measures enacted in China in 2020—including movement restrictions and social distancing—likely influenced brucellosis reporting through several indirect pathways, such as reduced human contact with infected animals, altered healthcare-seeking behaviors, and reallocation of surveillance resources toward emerging infectious diseases. Supporting this trend, a study in the Aksu region of Xinjiang documented a significant reduction in livestock brucellosis infection rates from 2017 to 2019.

From 2015 to 2023, human brucellosis incidence in Northern Xinjiang exhibited a significant positive spatial correlation, indicating spatial clustering and non-stationarity. This spatial heterogeneity can be attributed to regional variations in influencing factors [36].

Our Multiscale Geographically Weighted Regression (MGWR) analysis identified several statistically significant drivers, with varying magnitudes of influence as reflected in their regression coefficients. The most substantial protective factor was Gross Regional Product (GRP), with a mean coefficient of −0.335 (*p* < 0.05), showing the strongest effect in Tacheng and Altay Prefectures. This suggests that economic development facilitates investment in public health infrastructure, health education, and improved living conditions, thereby mitigating transmission risks. Conversely, the year-end sheep population (NOS) emerged as the strongest risk factor, with a mean coefficient of 0.696 (*p* < 0.05), particularly in Bayingolin Mongol Autonomous Prefecture. This underscores the role of sheep as the primary reservoir of Brucella in this region, where higher population density directly increases human exposure risk—especially among herders and livestock workers through direct contact with infected animals or contaminated materials.

Beyond the dominant influences of economic status and sheep population, climatic factors also contributed, though to a lesser extent. Longer annual sunshine hours (ASH, mean coefficient = 0.542, *p* < 0.05) were associated with higher incidence, especially in Ili and Bortala. This likely serves as a proxy for behavioral exposure, as extended daylight prolongs outdoor herding and farming activities, increasing potential contact with contaminated environments. Similarly, lower annual minimum temperature (TMIN, mean coefficient = −0.273,* p* < 0.05) was a significant risk factor in Altay, Changji, Turpan, and Hami. This association may be attributed to the fact that colder and more humid conditions favor the prolonged environmental survival of Brucella [37, 38].

Notably, several variables—including beef production (BP), mutton production (MP), annual maximum temperature (TMAX), and year-end cattle population (NOC)—did not demonstrate statistically significant associations with brucellosis incidence in the MGWR model (*p* > 0.05). This indicates that their independent effects, after accounting for spatial influences of factors, such as sheep population and GRP, are not robust. For instance, the weak and non-significant effect of beef production (BP, mean coefficient = 0.180,* p* = 0.860) suggests that cattle-to-human transmission may be less direct or dominant compared with sheep-related transmission cycles in Northern Xinjiang. Similarly, the negligible effect of maximum temperature (TMAX, mean coefficient = 0.028, *p* = 0.743) implies that high temperatures alone are not a key driver; instead, the relationship between climate and brucellosis is better captured by minimum temperature and sunshine duration.

Synthesizing these findings, we propose targeted prevention strategies that prioritize the most significant and spatially heterogeneous risk factors. First, given the over-riding importance of the sheep population, control efforts should prioritize mandatory and regular testing and vaccination of sheep, especially in high-risk areas, such as Bayingolin Mongol autonomous prefecture. Second, the strong protective role of GRP highlights the need to direct economic development gains toward strengthening veterinary services and public health infrastructure in less developed counties. Effective prevention and control strategies should account for regional differences and integrate measures to prevent contact transmission, address foodborne routes, enhance laboratory biosafety, support active surveillance through economic investment, and implement vaccination alongside selective culling [39, 40]. Finally, public health education should be tailored to local climatic conditions—emphasizing reduced prolonged indoor animal contact in colder regions and promoting protective measures during extended outdoor work in areas with abundant sunshine.

### Limitations

This study has several limitations. First, the reliance on passively monitored surveillance data from the CDC may introduce under-reporting and misclassification, potentially leading to an underestimation of true incidence. Of particular concern is the possibility of spatially non‑random under-reporting—for example, a higher likelihood of missed cases in remote areas with limited diagnostic capacity—which could bias both the spatial clustering analysis and the coefficient estimates in the MGWR model. Second, due to the lack of systematically available spatial demographic data on known high‑risk occupational groups, such as veterinarians and slaughterhouse workers, these factors were not included as explanatory variables. Their omission may leave residual spatial structures unaccounted for. To enhance the model’s applicability and explanatory power, future studies should develop more comprehensive data sets that incorporate these dimensions through targeted field surveys and integration of sector‑specific employment records.

## Conclusion

In conclusion, this study reveals significant temporal fluctuations and pronounced spatial heterogeneity in human brucellosis incidence in Northern Xinjiang between 2015 and 2023. Through the application of the MGWR model, we have identified spatially varying impacts of key drivers, including animal husbandry output, livestock populations—particularly sheep—beef production, and climatic factors, such as temperature and sunshine hours. These findings highlight that brucellosis dynamics are not uniform but are shaped by localized economic and environmental conditions. To translate these insights into meaningful public health outcomes, control strategies must be geographically precise and sector-specific. Prioritizing high-risk regions and occupational groups with targeted interventions—as emphasized throughout this study—is essential for effectively reducing the brucellosis burden in Northern Xinjiang and advancing toward its eventual elimination.

## Supplementary Information

Supplementary material 1. Visualization of MGWR model results.

Supplementary material 2. VIF value.

## Data Availability

The data supporting the findings of this study are available from the corresponding author upon reasonable request (Jiangshan Zhao, E-mail: zjscdc@163.com).
