# Supplementary material for: Assessing the spatiotemporal dynamics and driving factors of human brucellosis in Northern Xinjiang, China (2015–2023)
Source: Trop Med Health. 2026 Jan 17;54:20. doi: 10.1186/s41182-026-00899-6 (PMC12829208; doi:10.1186/s41182-026-00899-6)
Supplement: Supplementary file 1 — Supplementary material 1. Visualization of MGWR model results. [file 41182_2026_899_MOESM1_ESM.docx]

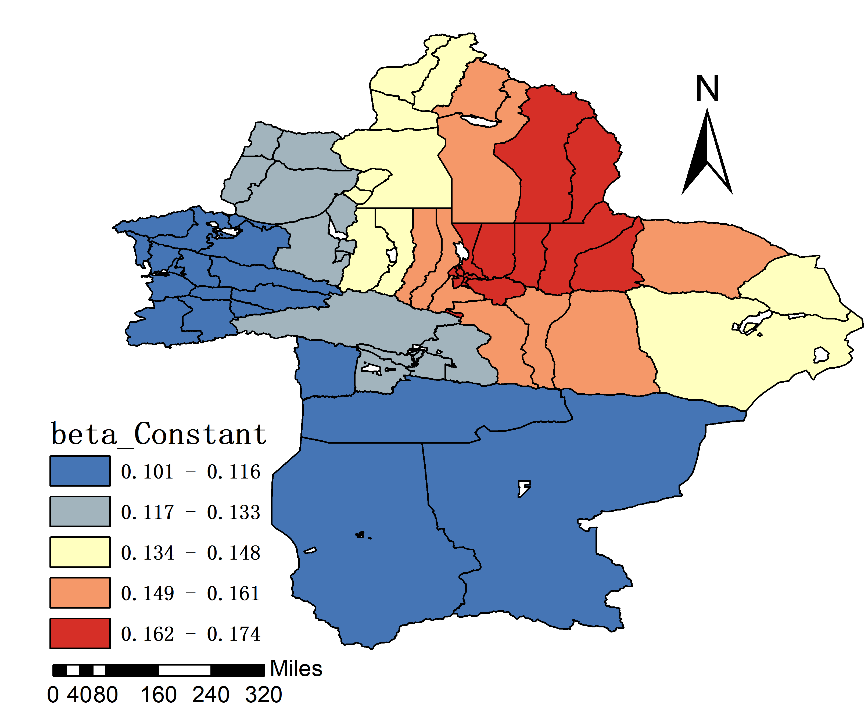


Figure S1. Constant term regression coefficients


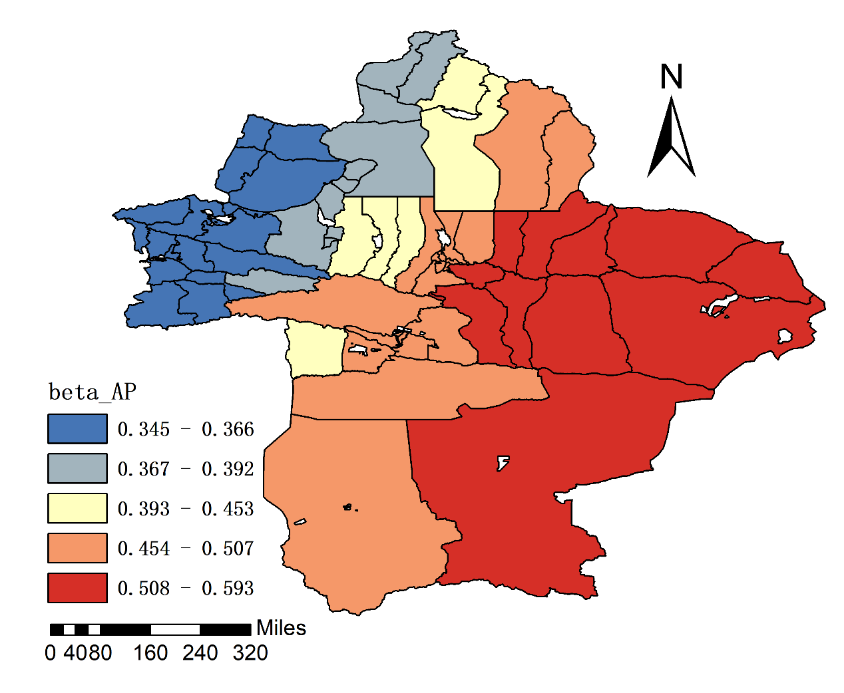


Figure S2. Spatiotemporal distribution of the NOS regression coefficients


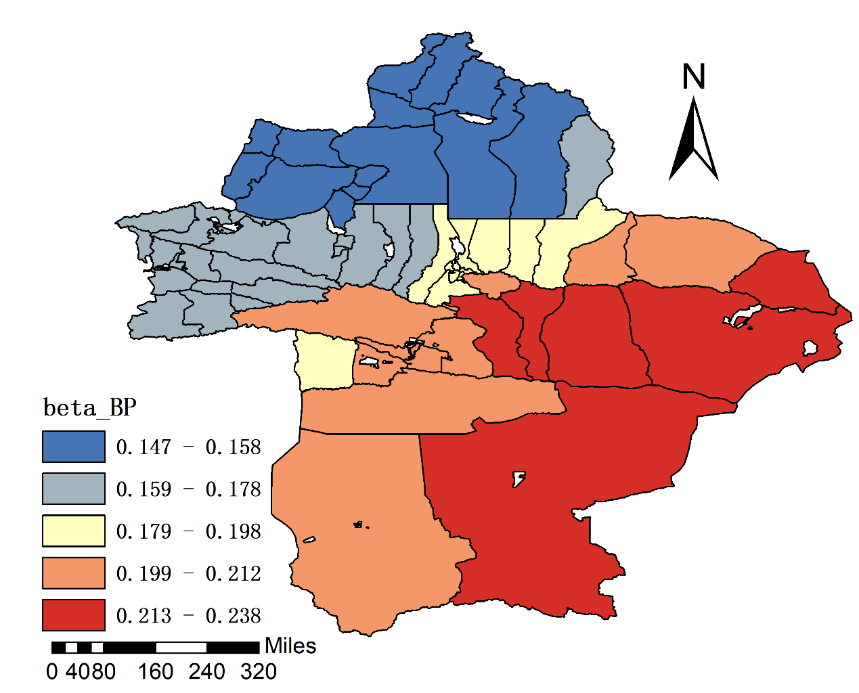


Figure S3. Spatiotemporal distribution of the BP regression coefficients


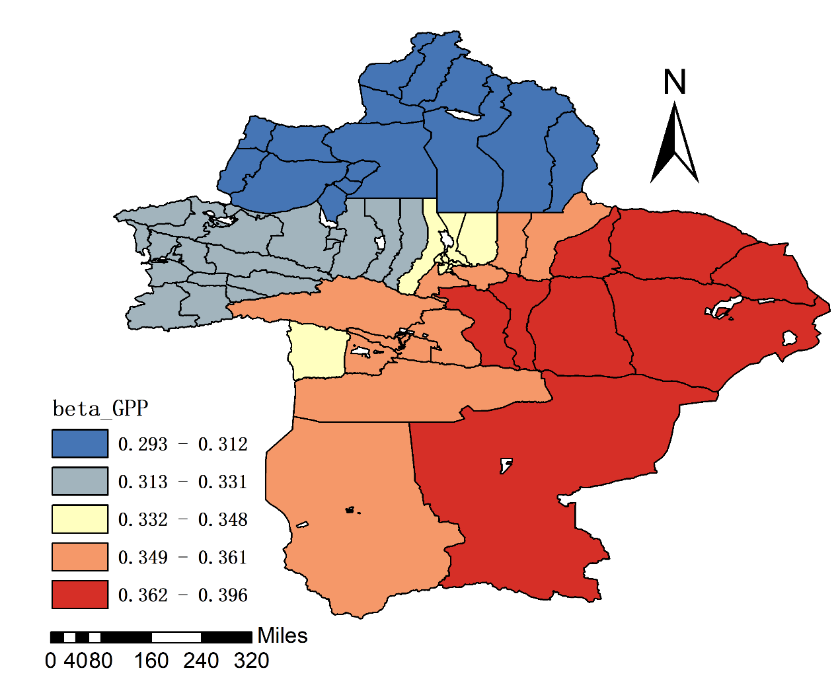


Figure S4. Spatiotemporal distribution of the GPP regression coefficients


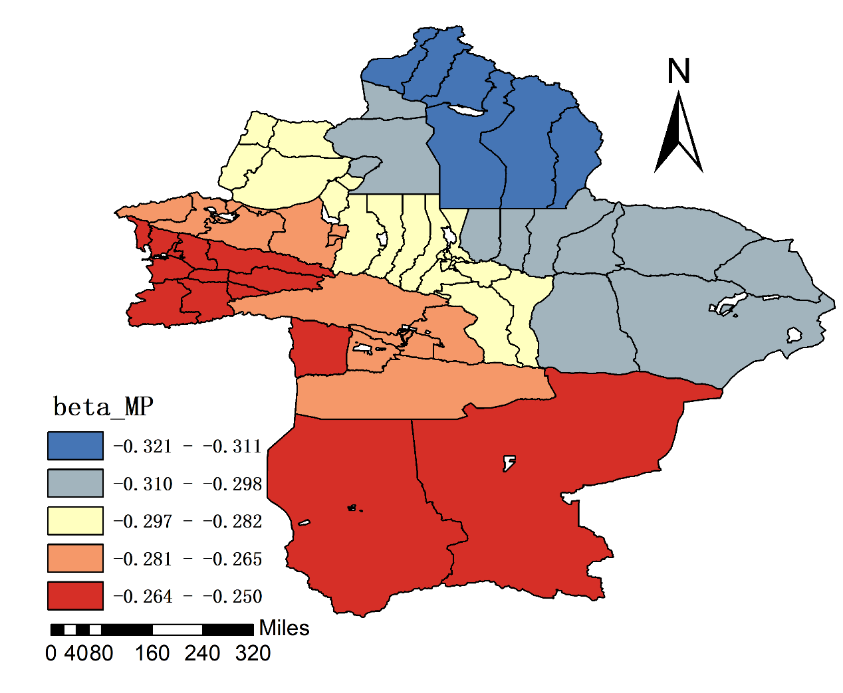


Figure S5. Spatiotemporal distribution of the MP regression coefficients


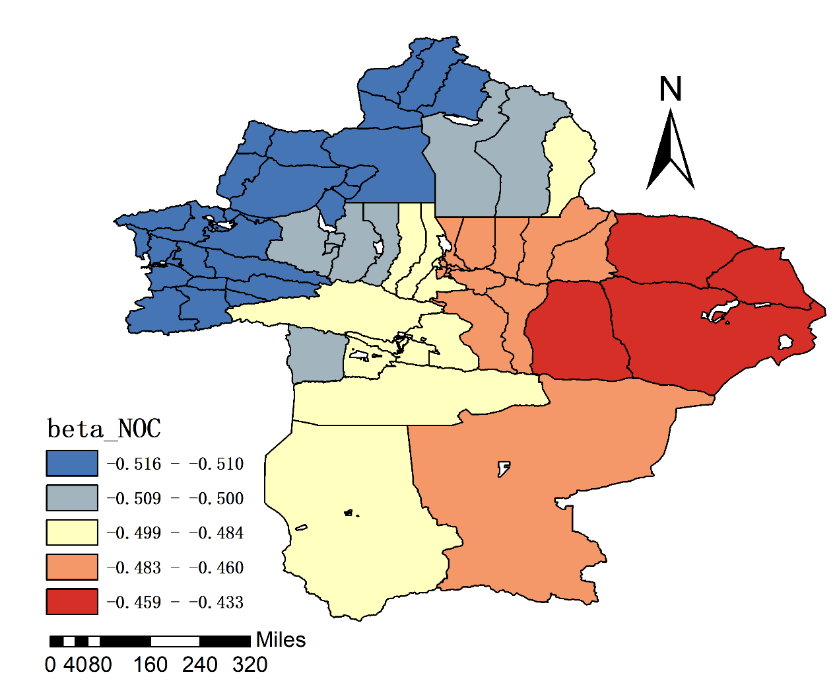


Figure S6. Spatiotemporal distribution of the NOC regression coefficients


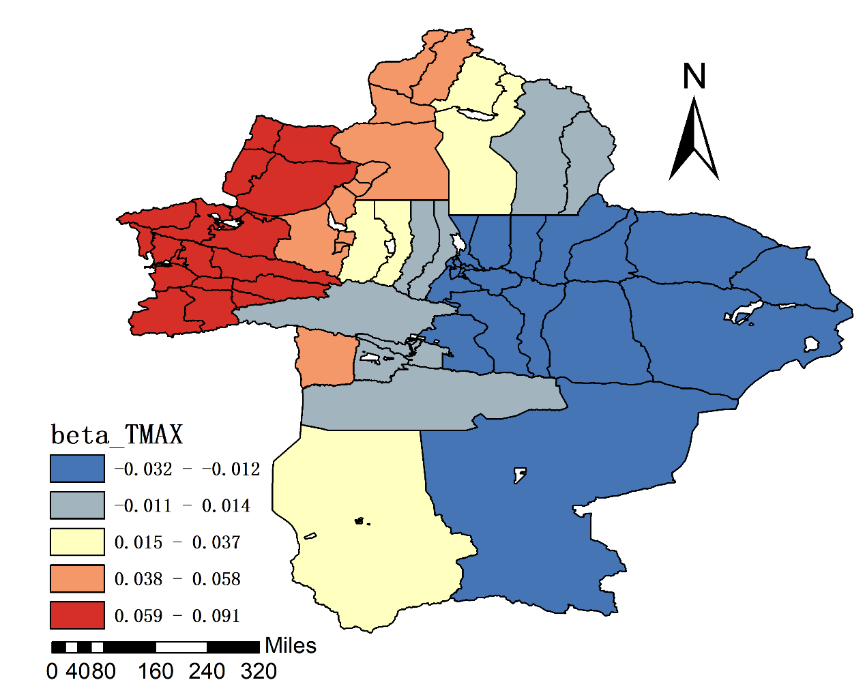


Figure S7. Spatial and temporal distribution of the TMAX regression coefficients
